# Using Artificial Intelligence to Obtain More Evidence? Prediction of Length of Hospitalization in Pediatric Burn Patients

**DOI:** 10.3389/fped.2020.613736

**Published:** 2021-01-18

**Authors:** Julia Elrod, Christoph Mohr, Ruben Wolff, Michael Boettcher, Konrad Reinshagen, Pia Bartels, German Burn Registry, Ingo Koenigs

**Affiliations:** ^1^Department of Paediatric Surgery, University Medical Centre Eppendorf, Hamburg, Germany; ^2^Burn Unit, Plastic and Reconstructive Surgery, Department of Paediatric Surgery, Altona Children's Hospital, Hamburg, Germany; ^3^Neoglia LTD, London, United Kingdom; ^4^German Society for Burn Treatment, Deutsche Gesellschaft für Verbrennungsmedizin, Committee of the German Burn Registry, Berlin, Germany

**Keywords:** artificial intelligence, burns, length of hospitalization, prediction, accuracy, paediatric

## Abstract

**Background:** It is not only important for counseling purposes and for healthcare management. This study investigates the prediction accuracy of an artificial intelligence (AI)-based approach and a linear model. The heuristic expecting 1 day of stay per percentage of total body surface area (TBSA) serves as the performance benchmark.

**Methods:** The study is based on pediatric burn patient's data sets from an international burn registry (*N* = 8,542). Mean absolute error and standard error are calculated for each prediction model (rule of thumb, linear regression, and random forest). Factors contributing to a prolonged stay and the relationship between TBSA and the residual error are analyzed.

**Results:** The random forest-based approach and the linear model are statistically superior to the rule of thumb (*p* < 0.001, resp. *p* = 0.009). The residual error rises as TBSA increases for all methods. Factors associated with a prolonged LOS are particularly TBSA, depth of burn, and inhalation trauma.

**Conclusion:** Applying AI-based algorithms to data from large international registries constitutes a promising tool for the purpose of prediction in medicine in the future; however, certain prerequisites concerning the underlying data sets and certain shortcomings must be considered.

## Background

Severe thermal injuries in the pediatric population generally have a far-reaching impact on those affected, their families, and society. Treatment costs of pediatric burns exceeded 211 million US dollars in the United States in 2000, not including the indirect economic impact related to long-term treatment of physical disability, care for psychological issues, absence at school, and the resulting lost wages (1). Severe burns are associated with long hospital stays (2). Prediction of length of stay (LOS) in the hospital is of great interest. It is not only important for counseling the affected families but also it has major implications for the management of health resources, capacity planning, and quality assurance in large burn (3–5). Hospitalization time might serve as a good indicator for injury-related morbidity and the incidence of clinical complications (6, 7).

Commonly, the assumption of a linear relationship between the burned total body surface area (TBSA) and the LOS (1 day stay per 1% TBSA) is applied as a rough estimation of the predicted hospitalization time (8). This rule of thumb, originating from 1986 and initially serving as a mean to contain hospitalization costs (9), is popular not only due to its simplicity but also due to its repeated validation with more recent data sets, as in Saffle et al. (10) using outcome data from the predecessor of the US National Burn Repository. However, some authors criticize the rule of thumb for its oversimplification (8, 11, 12). As an alternative, LOS is often predicted using multivariate models, as reviewed by Hussain and Dunn (7). Conversely, attempts to set up multivariate regression to predict LOS separately and systematically for the pediatric population are rare. In 1983, Bowser et al. (13) generated a regression equation based on 444 pediatric subjects, yielding only two independent variables, i.e., TBSA and the percentage of full-thickness burn to contribute significantly to the prediction of LOS.

The aim of this project was to close this gap using a linear regression analysis and the artificial intelligence (AI)-based prediction method random forest for the prediction of LOS in the pediatric population.

## Materials and Methods

Outcome data from pediatric burn patients were obtained from the Web-based international burn registry of German-speaking countries (Germany, Switzerland, and Austria) from the German Society for Burn Treatment (DGV) including the years 2015–2018. Institutions contributing to data entry—located in Germany, Austria, and Switzerland—are listed on the society's website (14). The manuscript is released in accordance with the publication guidelines of the German Burn Registry (VR-DGV-Project-ID: 2018-009).

Three methods to predict LOS were applied and compared in terms of their predictive capacity. Significant predictors of prolonged stay were also identified. In addition, predictive accuracy was calculated separately for patients with burns ≤ 20% TBSA only due to the low case numbers in patients with large % TBSA (196/8,542 patients = 2.29%). The choice of the parameters extracted from the register was adopted from those commonly found in adults: TBSA itemized by degree of burn, age, gender, inhalation trauma/injury (IHT), cause of injury, treatment in the (burn) intensive care unit (BICU), and ventilation. However, the parameters of treatment in the BICU and ventilation had to be eliminated due to incomplete and inconclusive data. Data quality was ensured by removing data demonstrating any of the following exclusion criteria: incomplete data for the above-named variables; data entry errors resulting in a contradiction with respect to the cause of burn, TBSA, or LOS. All calculations were performed using R Core Team (15).

### Rule of Thumb

The rule of thumb method is equivalent to a linear regression model with the TBSA coefficient equal to one, the coefficients of all other factors equal to zero, and a y-intercept of zero. This heuristic of expecting 1 day of hospitalization for each percentage of body surface area burned served as a benchmark in terms of its predictive accuracy.

### Multiple Linear Regression Model

In the multiple linear regression model, the R-based package “stat” was used, and no regularization was applied to this linear model assuming a Gaussian distribution over all factors. LOS was assumed to be the dependent variable, and the other parameters were applied as independent variables. The linear regression finds the optimal parameters for the equation:

$$\begin{array}{l} \text{HOSPITAL\_DAYS}=\text{c\_}0+\text{c\_}1^{*}\text{CAUSE\_BURN}\\ +\text{c\_}2^{*}\text{CAUSE\_SCALD}+\text{c\_}3^{*}\text{IHT}+\text{c\_}4^{*}\text{AGE}\\ +\text{c\_}5^{*}\text{SEX}+\text{c\_}6^{*}\text{DEGREE\_}2\text{a}+\text{c\_}7^{*}\text{DEGREE\_}2\text{b}\\ +\text{c\_}8^{*}\text{DEGREE\_}3 \end{array}$$

where c_0 is a constant determined for our regression model, and all remaining c_1 to c_7 quantify the contribution of this factor to the prediction of the dependent variable, i.e., LOS.

### Random Forest

Random forest is an advanced method of regression that can capture non-linear relationships between observed factors with lower variance than a single regression tree by averaging the prediction of multiple decision trees (16). Here, the R package randomForest (17) was applied and 150 different trees were used, of which each tree is trained on a different bootstrapped dataset. Bootstrapping is simply sampling from the original data with replacement (18). Each decision tree consists of edges that are conjunctions on a single variable being greater than or less than some value. In this way, each node of the tree divides the data into two subsets with the goal of making each subset more homogeneous. Which terminal node (leaf) of the tree a certain data point falls into decides the predictions by majority selection of the training data.

### K-Fold Cross Validation

For all three methods of prediction, both the mean absolute error (MAE) and the mean squared error (MSE) were calculated. For all models, k-fold cross validation (19) was performed, where *k* = 20 in our case, to estimate the generalization error in the future of real-world use. For this purpose, the data sets were split into 20 parts, and each model was trained 20 times. Each time, 19 parts served as training data. The performance of the model to predict LOS was then evaluated by applying it to the test set, i.e., the remaining one part. That way, a variance on how much test error fluctuates when the algorithm sees different subsets of the data during training is obtained. Results concerning MAE, respectively, MSE are reported as means over the 20 calculations. The performance of the three models was compared using a paired *t-*test. Prior to that, normality of the data was verified using the Kolmogorov–Smirnov test.

### Total Body Surface Area to Residual Plot

Cross validation test data set's residuals were plotted against the TBSA to allow investigation of the relationship between a patient's TBSA and the residual. A residual is the difference between the observed and the estimated value.

## Results

### Demographics and General Injury Data

Between 2015 and 2018, 8,915 children and adolescents were included in the registry. After removal of the deceased patients and inconclusive and missing data sets, 8,542 complete sets of patients with burns remained in the main analysis. Of these, 4,955 patients (58.00%) were boys. The mean age was 3.41 ± 4.45 years (range 0–18 years); the majority, 5,297 (62.01%), were children between 0 and 5 years of age. The most common causes of injury in descending order were scald (6,327 cases, 74.07%), burn (2,082 cases, 24.37%; among them, 974 were contact burns, 624 flame burns, 272 fat burns, and 136 burns due to explosions, including double entries), other (83 cases, 0.97%) and electricity (50 cases, 0.58%). A total of 52 children (0.61%) suffered from an inhalation injury. Mean LOS was 7.39 ± 8.51 days (range: 1–133 days). Mean TBSA was 5.79 ± 5.94% (range 0.1–87% TBSA).

**Figure 1:**
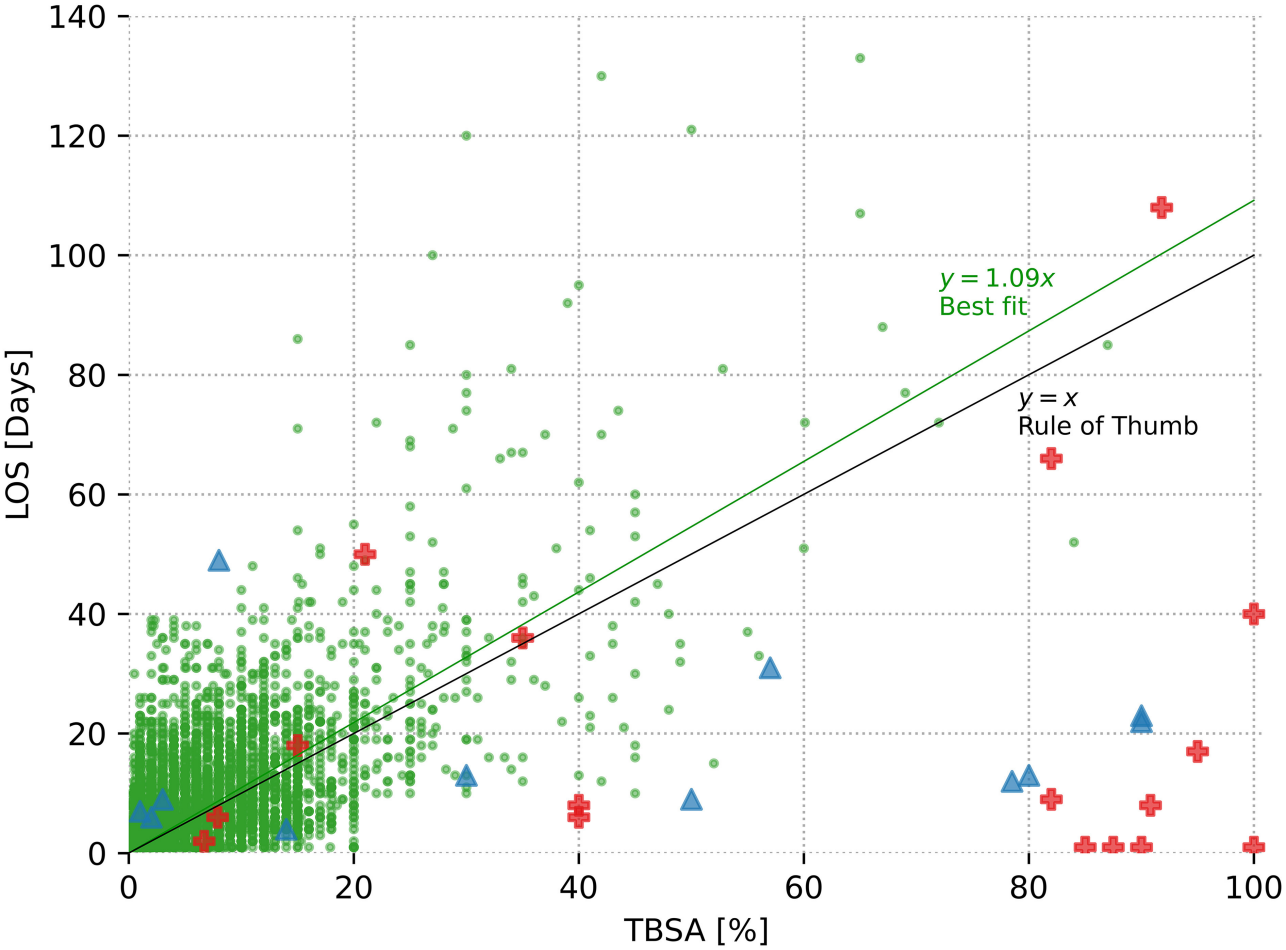
Relationship between length of stay and total body surface area (TBSA) affected. The 8,542 surviving burn subjects are depicted as green dots, and a best-fit line with a slope of m = 1.092 is displayed, predictive power *R*^2^ = 0.38; 17 deceased burned victims are displayed as red crosses (*R*^2^ = 0.02), and the 12 patients with dermatological conditions similar to superficial burns (toxic epidermal necrolysis, Stevens–Johnson syndrome, or staphylococcal scaled skin syndrome) are represented as blue triangles (*R*^2^ = 0.03). In addition, the rule of thumb (x = y) is displayed, yielding a predictive power of *R*^2^ = 0.35.

Furthermore, a total of 17 patients (11 males) had died (see red crosses in Figure 1). In this group, mean age was 12.12 ± 5.58 years (range 0–18 years). Mean TBSA was 62.92 ± 35.37% (range 6.7–100% TBSA). Inhalation injury occurred in nine (52.94%) of these 17 patients. Thus, mortality rates in descending order by cause of injury in our complete cohort was electricity (8.00%), other (0.75%), burn (0.48%), and scald (0.03%). Death occurred at a mean time of 22.34 ± 28.61 days (range 1–108 days), with a median of 8 days.

Moreover, a total of 12 patients (10 males) did not sustain a thermal injury but instead a spectrum of dermatological conditions whose characteristics were similar to a superficial skin burn. This included toxic epidermal necrosis (TEN), Stevens–Johnson syndrome, or the staphylococcal scaled skin syndrome (SSS). Here, mean age was 7.58 ± 6.01 years (range 0–18 years). Mean TBSA was 41.96 ± 36.38% (range 1–90%), mean LOS was 16.50 ± 12.98 days (range 4–49 days). No death was observed.

Figure 1 provides an overview of all patients in terms of TBSA and LOS. For patients who survived the burn injury, this results in a best-fit line with a slope of *m* = 1.09 days per TBSA affected. This simple best-fit line describes 38% of the variability of the dependent variable LOS (*R*^2^ = 0.38). Conversely, regarding the deceased burn victims, *R*^2^ = 0.02, indicating the lack of an obvious relationship between the time of death and the TBSA in this group. Finally, in the group of patients with dermatological conditions similar to burns, all patients survived and were discharged at a maximum of 49 days despite large TBSA involved with a similarly low *R*^2^ of 0.03. The rule of thumb suggests a linear relationship between TBSA and LOS, with an intercept of 0 and a slope of *m* = 1. One percentage of TBSA is equivalent to a hospital stay of 1 day.

The results from the linear regression model for LOS of the surviving burn patients are shown in Table 1. The model revealed cause scald, the presence of an inhalation injury, and TBSA by degrees 2a, 2b, and 3 to be statistically highly significant determinants of a prolonged hospital stay. In contrast, there was no significant relationship between cause burn, age, and gender and LOS in this group.

**Table 1:** Results of multiple linear regression analysis of length of stay for pediatric patients.

**Coefficient**	**Estimate**	**Standard error**	***T*-value**	***p*-value**
**Prediction of length of stay**				
Intercept	4.27	0.54	7.92	<0.001
Cause burn	−0.01	0.53	−0.01	0.991
Cause scald	−1.29	0.53	−2.44	0.015
Inhalation injury	3.46	0.85	4.09	<0.001
Age	0.00	0.02	0.24	0.814
Gender	−0.15	0.13	−1.14	0.260
Degree 2a	0.56	0.02	32.51	<0.001
Degree 2b	0.74	0.02	34.58	<0.001
Degree 3	1.78	0.03	65.86	<0.001

This model thus results in the following regression equation and an associated power of *R*^2^ = 0.49:

$$\begin{array}{l} \text{HOSPITAL\_DAYS}=4.27--0.01^{*}\text{CAUSE\_BURN}\\ -1.29^{*}\text{CAUSE\_SCALD}+3.46^{*}\text{IHT}+0.00^{*}\text{AGE}\\ -0.15^{*}\text{SEX}+0.56^{*}\text{DEGREE\_}2\text{a}+0.74^{*}\text{DEGREE\_}2\text{b}\\ +1.78^{*}\text{DEGREE\_}3 \end{array}$$

Here, cause scald, cause burn, IHT, and gender have binary formats, whereas degrees 2a, 2b, and 3 are indicated as actual percentage (0–100) and the age of the patient is entered in years.

The random forest-based prediction technique reveals the strongest predictive power provided by the observed variable degrees 3, 2b, and 2a (data not shown) and a predictive power of *R*^2^ = 0.49.

**Figure 2:**
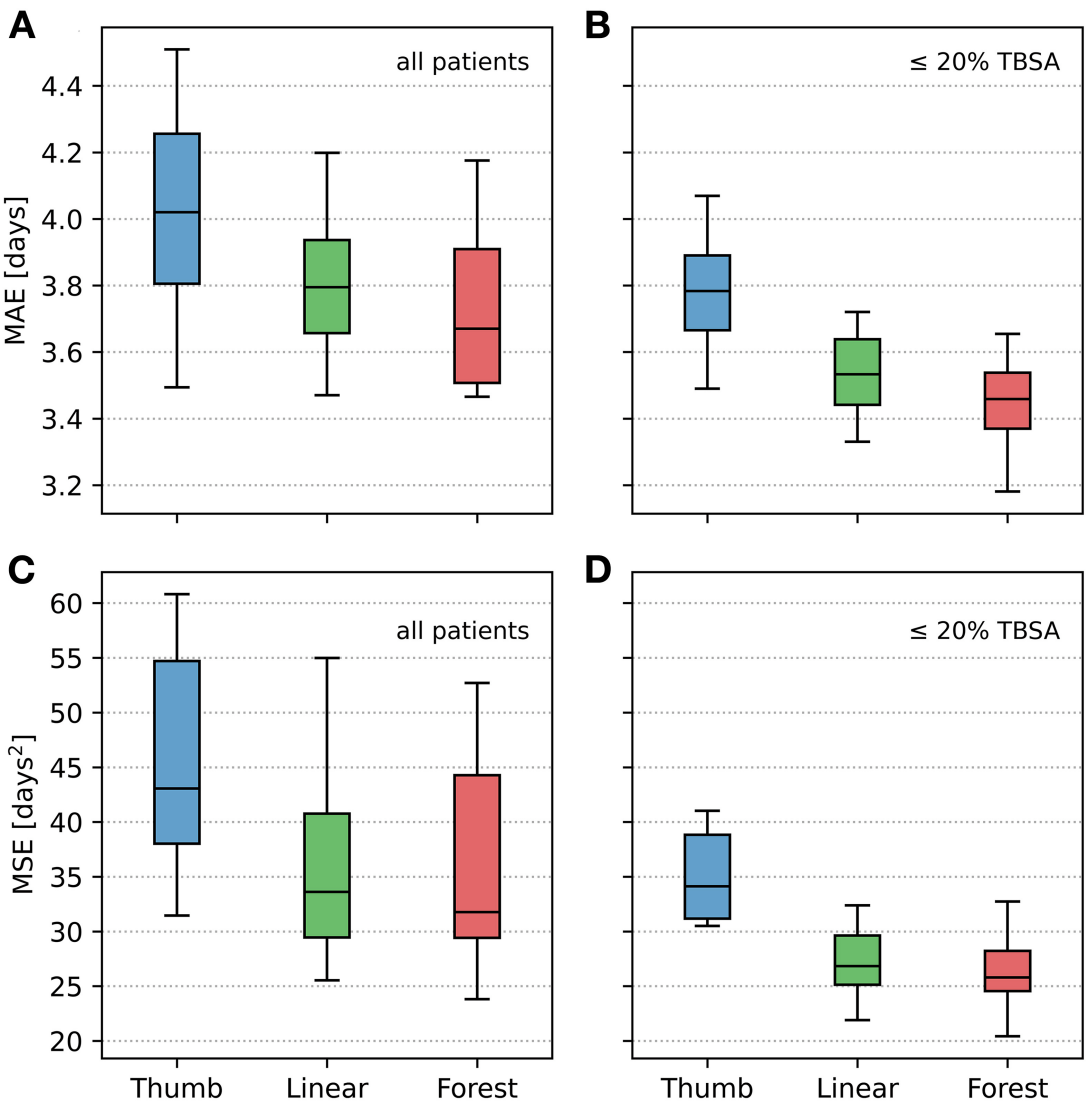
Comparative illustration of mean absolute error (MAE) and mean square error (MSE) using the three methods of prediction of length of stay: rule of thumb (“Thumb”), linear regression (“Linear”), and random forest (“Forest”). **(A,C)** All patients included into the analysis. **(B,D)** Sub-cohort including patients with injuries ≤ 20% TBSA only. The box plots display the 20 mean absolute error (MAE), respectively, 20 mean squared error (MSE) generated when running the models over each test set. The median is represented by the center line, the top and bottom of the box represent a 50% percentile around the median, and the outside lines represent a 95% percentile of the data.

The Kolmogorov–Smirnov test revealed all data concerning MAE and MSE to be normally distributed (*p* > 0.05). K-fold cross validation was performed to compare the effectiveness of the different LOS prediction techniques with the benchmark (effectiveness of the rule of thumb). In addition, these analyses were conducted separately for patients with injuries ≤ 20% TBSA. The results are depicted in Table 2 and Figure 2; significance levels are shown in Table 2. The random forest model results in the lowest MAE followed by the linear regression model and finally the rule of thumb reveals the largest MAE (Table 2), both for all patients and for the sub-cohort including patients with ≤ 20% TBSA only. Paired *t-*test analysis merely reveals the rule of thumb to be significantly inferior to the other two methods, whereas the difference in predictive accuracy between the linear model and the random forest-based approach is not substantial. Importantly, the terms MAE and MSE apply to the mean of all errors resulting from the 20 test sets, i.e., they are estimations of the generalization error.

**Table 2:** Estimated generalization errors of prediction of length of stay expressed as the mean absolute error (MAE) and mean square error (MSE) and comparison of effectiveness of each of the three models: rule of thumb (RT), linear regression model (LR), and random forest model (RF).

	**Wounds included**	**RT**	**LR**	**RF**	**RT vs. LR (*p-*value)**	**RT vs. RF (*p*-value)**	**LR vs. RF (*p*-value)**
MAE [d]	All	4.04	3.81	3.73	0.009	<0.001	0.262
	≤ 20% TBSA only	3.77	3.53	3.46	<0.001	<0.001	0.037
MSE [d^2^]	All	45.51	35.55	35.85	<0.001	0.007	0.907
	≤ 20% TBSA only	34.59	27.37	26.56	<0.001	<0.001	0.429

*Separate analysis for the sub-cohort of injuries ≤ 20% total body surface area (TBSA) is depicted. Pairwise t-test was performed to determine the significance (p-value) of the difference in predictive accuracy between the three methods*.

**Figure 3:**
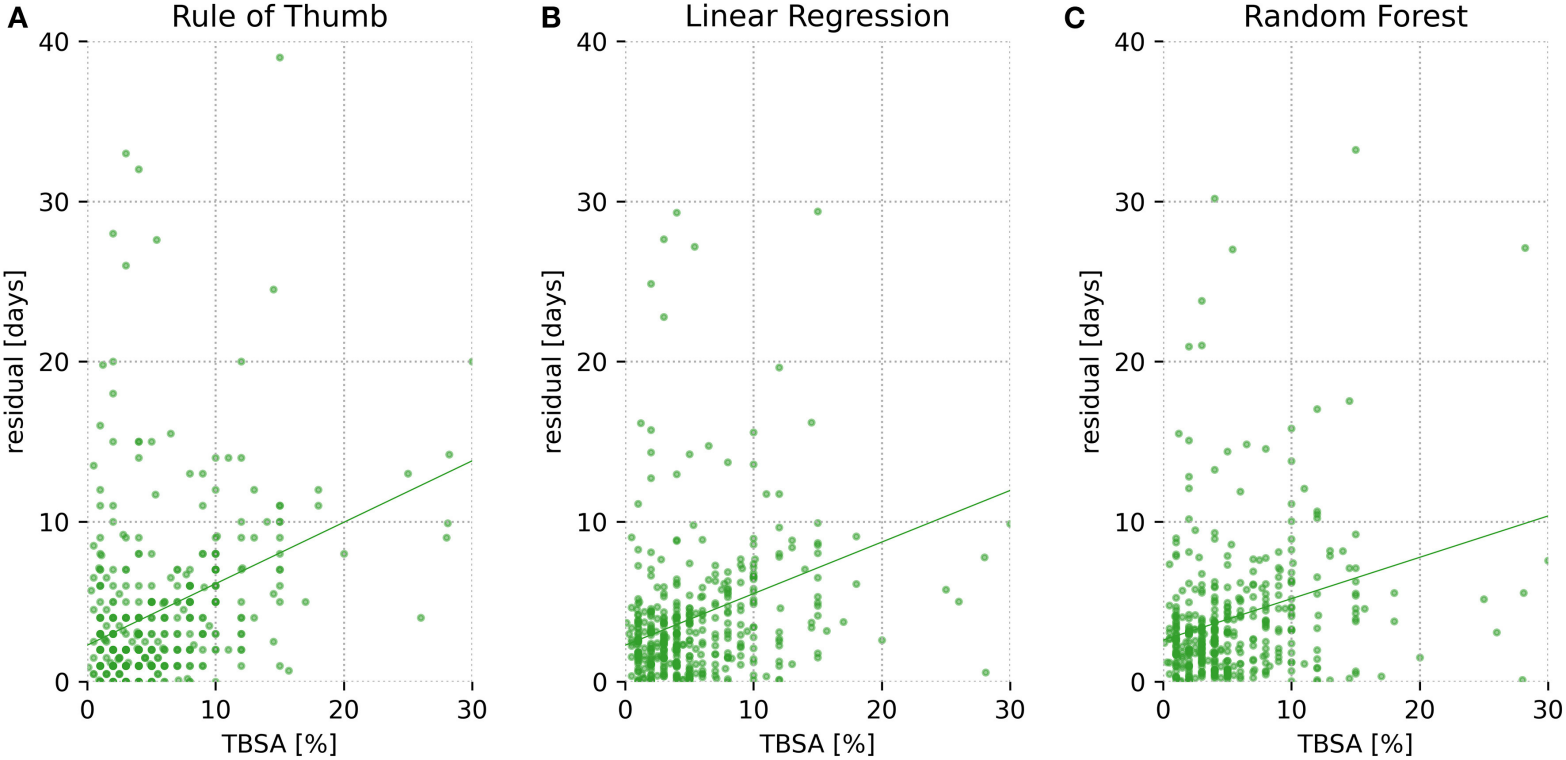
Residuals vs. total body surface area (TBSA) for all patients. Residuals are shown as a function of TBSA for one cross validation test set (1/20 of the data), depicted separately for each of the three models (**A** = Rule of Thumb, **B** = Linear Regression, **C** = Random Forest). Residuals are defined as the absolute difference between predicted and actual length of stay (LOS).

Cross validation test data set's residuals vs. TBSA were plotted for each of the three methods (total analysis only). The resulting Figure 3 reveals an increase in the residual error as TBSA increases for all three methods; random forest-based prediction depicts the flattest curve.

## Discussion

The rule of thumb is most convincing due to its absolute simplicity, assuming a hospitalization stay of 1 day per 1% TBSA (9), allowing for fast mental prediction of LOS in clinical everyday life. For the present data set, creating a significantly more accurate but equally simple formula, e.g., prediction LOS as a function of TBSA only, is not possible, as shown in Figure 1, in which the best-fit line nearly matches the rule of thumb. It is clear that any type of more complex prediction of LOS is only achieved at the expense of this simplicity.

Several studies have investigated whether the target of 1% per day could be achieved in different settings and come to diverging conclusions. A review by Saffle et al. (10) revealed a high consistency with the target, whereas others observed the actual LOS to be considerably higher than the target value for a large percentage of the patients (12, 20, 21). Alternatively, multiple regression is used for prediction of LOS (7), and with regard to AI, for example, model tree-based regression and support vector machine regression have been applied in the adult population by Yang et al. (4), indicating the AI-based techniques to be more effective than linear regression.

AI-based algorithms constitute a potentially powerful tool, as shown recently in several medical fields (22, 23), as they process huge amounts of data within seconds, resulting in predictions without any assumptions, yielding new patterns and connections. As to evaluate the true usefulness of an AI-based approach for the present application, it was indispensable to systematically test the performance of all three methods of interest on the same data set. The present analysis is based on 8,542 data sets provided by the German Burn Registry. In doing 20-fold cross validation, we get an evaluation of the variance of each estimator and, as a result, show the differences between each estimator as would be expected on new real-world data (Figure 2). The analysis depicts the linear regression model and the random forest-based approach to perform significantly better than the rule of thumb (Table 2). Nevertheless, it is striking that the random forest-based approach does not greatly outperform conventional methods of prediction.

As illustrated in Figure 3, prediction error increases for all methods with growing TBSA. This relationship is not surprising, since first, as with larger TBSA, the true hospitalization stay increases and hence there is greater room for error. Second, because large full-thickness wounds, unlike small injuries, are in many cases not primarily treated with conventional methods such as prompt split-thickness skin grafts (STSGs) but instead more complex treatments are used involving, for example, dermal regeneration templates with secondary coverage with STSGs after several weeks (24) or cultured epithelial autografts (25). These methods not only are lengthy but also render prediction of LOS difficult due to their susceptibility to infections (26) or the risk of rejection and prolonged wound healing (27). Third, extensive deep dermal or full-thickness burns lead to complex physiological derangements including sepsis-induced immunosuppression and consecutive (multi-)organ failure (28), which can have unforeseeable effects on LOS.

The fact that a separate analysis including injuries ≤ 20% TBSA only (Figure 2) tends to reveal a clearer distinction between the AI and the conventional methods might stem from the fact that AI-based methods are particularly dependent on the availability of high case numbers, indicating a limitation of the utility of AI-based methods in the upper range of percent of TBSA in the present study.

While the results of this study reveal that the immediate use of AI in day-to-day clinical practice is somewhat limited, one should keep in mind that even a modest increase in accuracy is of potential relevance for specific purposes, such as in the context of large-scale capacity planning and nationwide management of health resources. The sum of the slight enhancements in predictive accuracy in very large numbers of patients adds up to a relevant number of hospital days. Moreover, a more complex AI-based estimate of LOS could have a potential benchmarking function within the German Society for Burn Treatment, allowing for comparison of outcome quality (5).

Unlike the random forest model, which allows for intricate data fitting but is not intuitively understood, the linear method can be interpreted easily, generating a formula reflecting the contribution of each independent variable on the predicted variable. In the present analysis, its most influential factors were cause scald, IHT, and TBSA itemized by degree of burn, leading to a predictive power of *R*^2^ = 0.49. Results are similar to those of a previously published retrospective multivariate linear regression including children by Bowser et al. (13), revealing the combination of TBSA and the percentage of third-degree burn to be the most important variables, resulting in a predictive power of 0.59. In the adult population, a review by Hussain and Dunn (7) reveals age and percent of TBSA to be the strongest predictors; other important variables were percentage of the burn itemized by depth, burn, IHT, age, female gender, and performance of escharotomy. Interestingly, our linear regression model—including pediatric and adolescent patients only—did not reveal age to be of any importance.

For very large burn centers interested in the prediction of LOS by means of linear regression, it might be more reasonable to determine center-specific coefficients of the linear regression equation instead of simply assuming the formula proposed in this study. However, sample size requirements have to be met (29) and will probably impede this approach in most small- to middle-sized burn centers due to their moderate number of (severely) burned patients. In this case, we suggest applying the proposed formula.

Why is prediction of LOS so difficult in this population? One major difficulty is certainly that the actual hospital stay of an individual patient is not merely dependent on injury characteristics, patient demographics, preexisting medical conditions, and treatment strategies such as the use of modern dressings, which tend to allow early dismissal, but instead is also dependent on non-medical aspects. These comprise, among others, local and national hospital agreements, continuous changes in health insurance, as well as the local strategy concerning timing of transition of patients from the inpatient to the outpatient departments, and also possibly non-medical patient-related properties such as socioeconomic status (30–33). One limitation of the present study is the confined number of variables included in the German Burn Registry. Our work suggests an expansion of the number of observations. Furthermore, as shown in a survey in 17 burn centers in Germany, Switzerland, and Austria, each institution has its own standard operating procedure (SOP) regarding admission and treatment of severely burned patients (34). This lack of standardization certainly leads to differences in LOS. We cannot account for these factors, since, at present, treating institutions are anonymized in the burn registry, impeding adaptation of the prediction models for center-specific features. Another shortcoming of registry-based studies is that they tend to contain input errors due to human fuzziness. On this account, highly improbable cases were excluded from the present analysis; however, this also constitutes the risk of erroneous elimination of patients.

## Conclusion

In conclusion, this study presents the first systematic and comparative investigation of the accuracy of three distinct algorithms in the prediction of LOS in pediatric burn patients, including an AI-based approach in an international cohort. The results indicate a modest, however, statistically significantly improved accuracy of the random forest-based prediction of LOS in comparison to the commonly applied heuristic expecting 1 day of hospitalization for each percentage TBSA. Yet, expanding the total number of severely burned patients, the quantity of observed variables per patient, and non-medical information concerning the treating institution and its case and discharge management is indispensable as to further improve its accuracy. Concepts using AI should be evaluated in future prospective studies in burn patients whenever large, comprehensive international registries are available. Especially, after solving data protection issues, instantaneous, automated extraction of anonymized data from hospital information systems would enable improvement of AI-based algorithms for the purpose of obtaining more evidence in medicine.

## Data Availability Statement

The data analyzed in this study is subject to the following licenses/restrictions: Data was extracted from the burn registry of the German Society for Burn Treatment (DGV), Committee of the German Burn Registry, Luisenstrasse 58–59, 10117 Berlin, Germany. On demand, data can be requested.

## Author Contributions

All authors substantially contributed to the present article and are legally responsible for the content. JE: concept and design of the work, analysis, interpretation of a data, statistics, drafting the work, and revision of the work. CM: literature research, planning, statistics, and revision of the work. RW: planning, statistics, and revision of the work. MB: data analysis and plotting, data interpretation, and final revision of the work. PB: drafting the work and revision of the work. KR: analysis, interpretation of a data, statistics, drafting the work, and revision of the work. IK: substantial contribution to the concept and design of the work, analysis, interpretation of a data, statistics, drafting the work, and revision of the work. German Burn Registry: acquisition of the data, providing the infrastructure of the registry, and revision of the work by the internal review board.

## Conflict of Interest

RW was employed by the company Neoglia LTD. The remaining authors declare that the research was conducted in the absence of any commercial or financial relationships that could be construed as a potential conflict of interest.
